# [18F]FDG-PET/CT Radiomics for Prediction of Bone Marrow Involvement in Mantle Cell Lymphoma: A Retrospective Study in 97 Patients

**DOI:** 10.3390/cancers12051138

**Published:** 2020-05-02

**Authors:** Marius E. Mayerhoefer, Christopher C. Riedl, Anita Kumar, Ahmet Dogan, Peter Gibbs, Michael Weber, Philipp B. Staber, Sandra Huicochea Castellanos, Heiko Schöder

**Affiliations:** 1 Department of Radiology, Memorial Sloan Kettering Cancer Center, New York, NY 10065, USA; riedlc@mskcc.org (C.R.); gibbsp@mskcc.org (P.G.); huicochs@mskcc.org (S.H.C.); schoderh@mskcc.org (H.S.); 2 Department of Biomedical Imaging and Image-guided Therapy, Medical University of Vienna, 1090 Vienna, Austria; michael.weber@meduniwien.ac.at; 3 Department of Medicine, Memorial Sloan Kettering Cancer Center, New York, NY 10065, USA; kumara2@mskcc.org; 4 Department of Pathology, Memorial Sloan Kettering Cancer Center, New York, NY 10065, USA; dogana@mskcc.org; 5 Department of Medicine, Medical University of Vienna, 1090 Vienna, Austria; philipp.staber@meduniwien.ac.at

**Keywords:** lymphoma, FDG, PET/CT, bone marrow

## Abstract

Biopsy is the standard for assessment of bone marrow involvement in mantle cell lymphoma (MCL). We investigated whether [18F]FDG-PET radiomic texture features can improve prediction of bone marrow involvement in MCL, compared to standardized uptake values (SUV), and whether combination with laboratory data improves results. Ninety-seven MCL patients were retrospectively included. SUVmax, SUVmean, SUVpeak and 16 co-occurrence matrix texture features were extracted from pelvic bones on [18F]FDG-PET/CT. A multi-layer perceptron neural network was used to compare three combinations for prediction of bone marrow involvement—the SUVs, a radiomic signature based on SUVs and texture features, and the radiomic signature combined with laboratory parameters. This step was repeated using two cut-off values for relative bone marrow involvement: REL > 5% (>5% of red/cellular bone marrow); and REL > 10%. Biopsy demonstrated bone marrow involvement in 67/97 patients (69.1%). SUVs, the radiomic signature, and the radiomic signature with laboratory data showed AUCs of up to 0.66, 0.73, and 0.81 for involved vs. uninvolved bone marrow; 0.68, 0.84, and 0.84 for REL ≤ 5% vs. REL > 5%; and 0.69, 0.85, and 0.87 for REL ≤ 10% vs. REL > 10%. In conclusion, [18F]FDG-PET texture features improve SUV-based prediction of bone marrow involvement in MCL. The results may be further improved by combination with laboratory parameters.

## 1. Introduction

Unilateral iliac crest bone marrow biopsy (BMB) is still a standard procedure in the majority of lymphomas, as bone marrow involvement is a criterion for stage IV disease [1]. With the exception of Hodgkin lymphoma, where [18F]FDG-PET/CT (positron emission tomography/computed tomography after injection of the radiolabeled glucose analogue 2-[18F]-fluoro-2-deoxy-D-glucose) replaces BMB [2,3], and diffuse large B-cell lymphoma (DLBCL), where no BMB is required for confirmation of focal FDG-avid bone (marrow) lesions on PET/CT [4,5], BMB is the recommended test to rule out bone marrow involvement in lymphoma patients [1,5]. This includes mantle cell lymphoma (MCL), which accounts for 7% of NHL cases, and shows bone marrow involvement in 55–90% of cases at the time of diagnosis [6]. Notably, MCL can have an aggressive or an indolent course [7], which directly affects glucose metabolism and FDG uptake [8]. Consequently, previous studies with small MCL cohorts have suggested that [18F]FDG-PET cannot reliably capture bone marrow involvement in MCL [9,10,11,12,13].

Radiomics is a computer-assisted technique to extract quantitative markers—the so-called radiomic features—from diagnostic medical images [14,15]. Radiomic features include texture features that capture spatial signal intensity (i.e., gray-level) patterns, and may therefore be used to assess heterogeneity [14,15,16]. It has been suggested that such radiomic features are linked to biological properties of cancers such as mutational burden, proliferation and aggressiveness [16,17,18,19,20]; contrary to histological biomarkers derived from biopsies, radiomics can interrogate the whole tumor volume across the entire body, rather than just a small sample from a single site [21]. The value of radiomic features extracted from [18F]FDG-PET for assessment of bone marrow infiltration in MCL has so far not been investigated. Only a single study in DLBCL has used [18F]FDG-PET radiomic features, and reported a good performance for disease prediction, as well as prognostic potential of this technique [22]. 

The goal of the present study was therefore to determine (1) whether [18F]FDG-PET radiomic texture features can improve prediction of bone marrow involvement in MCL patients, compared to traditional standardized uptake values (SUV); (2) whether the degree of marrow infiltration has an impact on the predictive value of radiomic features for assessment of bone marrow involvement; (3) whether combination of [18F]FDG-PET radiomic features and routine laboratory data, as recommended by guidelines for radiomic study design [23], can further improve prediction of bone marrow involvement; and (4) whether radiomic features can predict the Ki-67 proliferation index. 

## 2. Results

### 2.1. Patient Characteristics

Ninety-seven patients (31 women and 66 men; mean age, 63.5 ± 12.5 years) met our criteria for participation in the study (Table 1). Three patients (3.1%) were diagnosed with Ann Arbor stage I, eleven patients (11.3%) with stage II, 14 patients (14.4%) with stage III, and 69 patients (71.1%) with stage IV disease. [18F]FDG-PET/CT was performed using the Discovery STE scanner for 37 patients, the Discovery 690 scanner for 36 patients, the Discovery 600 for 18 patients, and the Discovery 710 scanner for six patients each. 

According to the pathology reference standard, bone marrow involvement was present in 67/97 patients (69.1%): 46/97 patients (47.4%) showed a relative bone marrow involvement (percentage of cellular bone marrow) (REL) > 5%; 41/97 patients (42.3%) showed REL > 10%; 43/97 patients (44.3%) showed an absolute bone marrow involvement (percentage of fatty + cellular bone marrow) (ABS) > 5%; and 35/97 patients (36.1%) showed ABS > 10% bone marrow involvement. Mean white blood count (WBC) was 10.5 ± 11.9 × 10^9^ /L, and mean lactate dehydrogenase (LDH) was 232.3 ± 86.0 U/L. Mean Ki-67 was 28.9 ± 23.7%; 33/67 (49.3%) of patients with bone marrow involvement showed a high Ki-67 index (≥30%). 

### 2.2. Prediction of Bone Marrow Involvement

Principal component analysis (PCA) reduced the information from the three SUVs and the 16 [18F]FDG-PET/CT texture features to five principal radiomic components (see Table S1), which constituted the radiomic signature. This radiomic signature was then used for the five pairwise comparisons—involved vs. uninvolved bone marrow; REL ≤ 5% vs. REL > 5%; REL ≤ 10% vs. REL > 10%; ABS ≤ 5% vs. ABS > 5%; ABS ≤ 10% vs. ABS > 10%; and Ki-67 < 30% vs. Ki-67 ≥ 30%.

**Figure 1:**
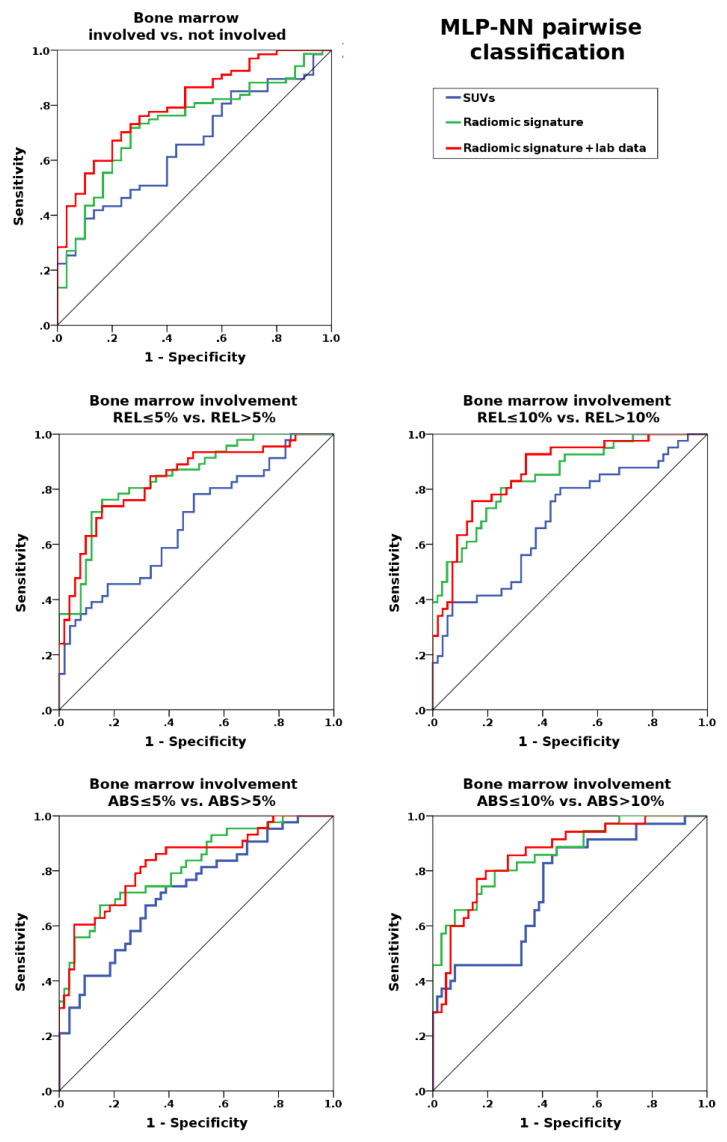
Areas under the receiver operating characteristic curve for standardized uptake values (SUVs) alone; the radiomic signature (SUVs and gray-level co-occurrence matrix (GLCM) features); the radiomic signature combined with laboratory data (WBC and LDH); for assessment of bone marrow involvement. Performance generally improves with the percentage of relative or absolute bone marrow involvement (REL and ABS), but more prominently for the radiomic signature, with and without combination with laboratory data.

Multi-layer perceptron neural network (MLP-NN)-based differentiation of involved from uninvolved bone marrow was unsatisfactory for both the [18F]FDG-PET radiomic signature and SUVs (maximum, mean, and peak) alone, with areas under the curve (AUCs) of up to 0.69 and 0.66, respectively; accuracies in training and test datasets also yielded similar results (Table 2, Figure 1). These results were clearly improved when laboratory data were added to the radiomic signature; using this combination, AUCs of up to 0.80 were achieved (Table 2, Figure 1).

**Figure 2:**
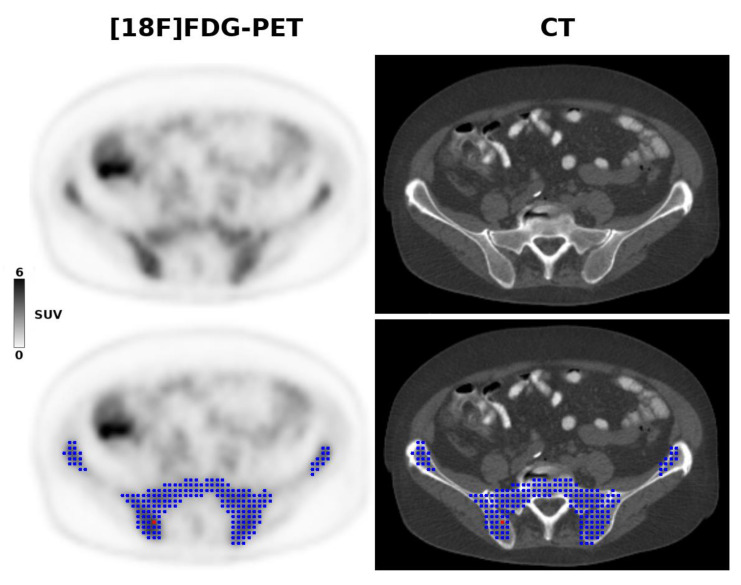
A 68-year-old patient with stage IV mantle cell lymphoma due to biopsy-proven bone marrow involvement. The [18F]FDG-PET 3D radiomic analysis is based on the metabolic tumor volume (blue) within the pelvis, constructed using the previously recommended 41% SUVmax threshold, and controlled by CT anatomy. The red dot shows the voxel with the highest SUV (i.e., the SUVmax).

By contrast, for REL ≤ 5% vs. REL > 5% involvement, the [18F]FDG-PET radiomic signature showed clearly better classification performance than SUVs alone, with AUCs of up to 0.82 vs. 0.68. The same trend was observed for REL ≤ 10% vs. REL > 10% involvement, with AUCs of up to 0.83 vs. 0.69, respectively, and also for ABS ≤ 5% vs. ABS > 5% (AUC 0.83 vs. 0.73) and ABS ≤ 10% vs. ABS > 10% (AUC 0.82 vs. 0.75) involvement. In all cases, addition of laboratory data improved the results achieved with the radiomic signatures, with AUCs of up to 0.87 (REL ≤ 5% vs. REL > 5%), 0.88 (REL ≤ 10% vs. REL > 10%), 0.83 (ABS ≤ 5% vs. ABS > 5%), and 0.82 (ABS ≤ 5% vs. ABS > 5%) (Figure 2). Respective accuracies in training and test datasets are provided in Table 2.

Finally, for low (<30%) vs. high (≥30%) Ki-67, the [18F]FDG-PET radiomic signature showed clearly higher AUCs (median, 0.80; range, 0.77–0.84) than the SUVs (median, 0.67; range, 0.65–0.67). In the training dataset, median accuracies were 70.8% (70.8–77.1%) for the radiomic signature, and 56.3% (56.3–60.4%) for the SUVs; whereas in the test dataset, median accuracies were 68.4% (63.2–78.9%) for the radiomic signature and 68.4% (57.9–68.4%) for the SUVs.

## 3. Discussion

According to the current International Working Group (IWG) consensus, unilateral iliac crest bone marrow biopsy—a procedure associated with anxiety and pain [24], and very rarely with complications such as bleeding, infection, and nerve damage—is mandatory for baseline staging of MCL [5], despite the fact that this test is prone to false-negative results in patients with focal lesions [1]. Our study results suggest that [18F]FDG-PET radiomic features carry potentially useful information for the non-invasive assessment of bone marrow involvement in MCL patients, and also, to some extent, proliferative activity as assessed by Ki-67 in clinical practice. While radiomic features were only of limited value when patients with minimal degrees of lymphomatous bone marrow infiltration were included, results were promising once 5% or 10% thresholds of (relative or absolute) marrow infiltration were applied. Here, the addition of [18F]FDG-PET-based texture features to standard SUVs clearly improved prediction of disease, with AUCs of up to 0.82 (REL > 5%) and 0.83 (ABS > 5%). A further improvement was achieved when the radiomic signature comprising [18F]FDG-PET texture features and SUVs was combined with WBC and LDH, two laboratory parameters routinely obtained in lymphoma patients, yielding AUCs of up to 0.87 (REL > 5%). Our findings therefore support the notion that (1) advanced radiomic features can add clinically relevant information to standard PET metrics; and that (2) an integrated diagnostic machine learning approach that combines information from different sources (e.g., PET and laboratory data) is preferable to a single-source approach.

Visual [18F]FDG-PET evaluation for detection of bone marrow involvement has proven unsatisfactory in MCL with reported sensitivities of 12%–52% [9,10,11]. Quantitative [18F]FDG-PET metrics for assessment of MCL bone marrow involvement were only investigated in a single study by Morgan et al., who suggested that voxel-wise analysis using a cut-off value of >38% of voxels with SUVmax < 0.95 could be clinically useful [25]. However, this study was clearly limited in terms of cohort size, with ten patients for SUV cut-off calculation and 16 patients for validation. For patients with follicular lymphoma, Adams et al. aimed to establish cut-off values for SUVmax, SUVmean, SUVpeak—the three traditional PET metrics that were also used in our study—to distinguish involved from uninvolved bone marrow, with unilateral iliac crest biopsy serving as the reference standard [26]. In their sample of just 22 patients, discrimination of involved and uninvolved bone marrow was successful using cut-off values of 2.1 for SUVmax, 1.3 for SUVmean, and 1.7 for SUVpeak, with AUCs of 0.89, 0.85 and 0.87, respectively; whereas visual assessment failed. In 41 follicular lymphoma patients, El-Najjar et al. applied a 2.0 average SUVmax threshold to [18F]FDG-PET/CT, and observed a sensitivity of 53% for prediction of diffuse bone marrow involvement that improved to 83% when the liver uptake was introduced as a reference with a 1.0 ratio threshold [27]. In a mixed population of 60 patients with Hodgkin and different types of Non-Hodgkin lymphoma, including only four cases of MCL, Asenbaum et al. reported AUCs of 0.87 for focal bone marrow involvement and 0.75 for assessment of diffuse marrow involvement, using SUVmax-ratios (bone marrow-to-liver uptake) with cut-off values of 95.25% and 70.2%, respectively [28]. Finally, in a population of 108 not further specified NHL patients, Öner et al. achieved an AUC of 0.65 using a 3.2 SUVmax cut-off. Neither of the above studies used a validation cohort to confirm their findings [29]. 

As the only study so far that used higher-order [18F]FDG-PET radiomic features for assessment of lymphoma bone marrow involvement, Aide et al. extracted co-occurrence and size-zone matrix features in addition to histogram features from PET images of 82 DLBCL patients [22]. Here, PET histogram features proved superior to higher-order texture features (with AUCs of 0.80–0.82 vs. 0.54–0.75) for assessment of diffuse bone marrow involvement, with the feature Skewness showing 82% sensitivity and specificity. Contrary to our study, no validation cohort was used, no combinations of multiple radiomic features, or radiomic features with clinical or laboratory parameters, were evaluated.

While our application of a 5% threshold for bone marrow infiltration had a considerable positive effect on the performance of the radiomics-based prediction model, a further elevation of the threshold to 10% only led to minor improvements. These findings suggest that, at least in MCL, very low volume marrow infiltration below the 5% threshold represents the main challenge for [18F]FDG-PET radiomics. However, our findings also suggest that beyond the 5% threshold, additional data from sources other than PET—in our case, laboratory data—may be needed for a more reliable, more accurate prediction of bone marrow involvement. Similar approaches were used in previous PET studies of different cancers. Lv et al. analyzed a series of 128 patients with nasopharyngeal carcinoma, and reported that PFS prediction was improved when the [18F]FDG-PET/CT radiomic signature was combined with platelet count, immunoglobulin A antibodies against EBV viral capsid antigen, and tumor stage, especially in the subgroup of 86 patients with local-regional advanced disease [30]. Mu et al. observed improved prediction of durable clinical benefit in non-small cell lung cancer when the pre-treatment [18F]FDG-PET/CT radiomic signature was combined with the ECOG clinical performance score and tumor stage in a sample of 194 patients scheduled to undergo immunotherapy [31]. Finally, we found that the addition of WBC, LDH, Ki-67 index, and ECOG status as an addition to pre-treatment [18F]FDG-PET radiomic features clearly improved 2-year PFS prediction in a previous study of 107 MCL patients [32]. Such multivariable analyses that provide a more holistic model of tumor biology are explicitly endorsed by Lambin et al., who list the combination of radiomic and non-radiomic data as a quality criterion within their radiomics score [23].

While our prediction model clearly requires further improvement, there are two main scenarios where application of a radiomics signature for bone marrow evaluation in MCL could be potentially useful. First, radiomics could complement staging in patients that are already in stage IV (e.g., due to GI tract involvement); here, bone marrow biopsy could possibly be omitted because it does not change staging, and an improved version of our radiomic signature would nevertheless provide sufficiently accurate information on the likelihood of bone marrow involvement. Second, as our evaluation of patients with high and low Ki-67 index suggests, [18F]FDG-PET radiomic features may provide information on cell proliferation, and could therefore be used for monitoring after treatment and possibly even as a prognostic marker. Contrary to bone marrow involvement per se, Ki-67 is recognized as a prognostic marker and is therefore also included in the MCL International Prognostic Index (MIPI-c), using a 30% cut-off [33].

The radiomics approach enables the calculation of a multitude of image-based quantitative features, ranging from simple first-order features such as mean, maximum and minimum values or percentiles to more sophisticated metrics that include texture features, model-based features, and transform-based features that rely on spatial relationships between the gray-level values (i.e., signal intensities) of pixels or voxels; and shape features that provide quantitative descriptors of geometric properties of objects such as tumors [34]. Features derived from the gray-level co-occurrence matrix (GLCM)—a second-order gray-level histogram—were chosen for our study because the GLCM is one of the best established and most widely used texture feature classes, and has also previously been applied to PET [20,30,31,32,35,36,37,38]. We chose not to include additional feature classes in order to keep the complexity of the prediction model as low as possible, and thus reduce the risk of overfitting, which is a common pitfall for radiomics studies. This was also the reason for our additional application of principal component analysis for further dimensionality reduction, as previously recommended [23]. The maximum number of seven features (five principal radiomic components and two laboratory parameters) as input for the neural network relative to the number of patients in the smallest group in this study (35 patients with ABS > 10% involvement)—i.e., a 1:5 ratio—was regarded as tolerable. Nevertheless, it is quite possible that radiomic features derived from other feature classes (e.g., the run-length or size-zone matrix) might have performed better than GLCM features, or that their exclusive use or addition may have further improved the prediction model. 

Similar to the study by Morgan et al., our image analysis was confined to the bony pelvis, for two reasons. First, MCL is a lymphoma subtype mainly found in elderly patients in whom degenerative changes of the spine, such as activated osteochondrosis with/without intravertebral disc herniations and spondylarthritis, are common. Since these degenerative conditions frequently lead to foci of increased, reactive or inflammatory FDG uptake, our decision to exclude the spine was an attempt to reduce the risk of generating PET radiomic data from non-lymphoma-related tracer accumulations. Second, since the biopsy reference standard was, and routinely is, obtained in the iliac crest, we hypothesized that radiomic features extracted from the same anatomic site would yield the best correlation with the reference standard. While it has been shown that PET as a whole-body technique can detect sites of bone marrow involvement missed by blind iliac crest biopsy [39], our retrospective studies design precluded the possibility of performing additional biopsies for histological verification in such cases of suspected “false-negative” biopsy/pathology results, which would have left us without a reference standard. 

There are several limitations to our study, the most obvious ones being the retrospective design and the modest cohort size; however, MCL is considered a rare lymphoma subtype, and this is the first study to evaluate the PET radiomics approach for assessment of bone marrow involvement in this particular lymphoma subtype. While the MLP-NN used for prediction of bone marrow involvement represents a well-established machine learning algorithm, deep learning techniques such as convolutional neural networks (CNN) might have performed even better. However, the higher intrinsic complexity of CNNs is better suited for the use with large multicentric data where their potential can be fully exploited, and where the risk of overfitting is lower than with our limited sample size [40].

## 4. Materials and Methods 

### 4.1. Patients and Design

Patients with histologically proven, treatment-naïve MCL, as diagnosed by a reference pathologist, who had undergone [18F]FDG-PET/CT for routine pre-therapeutic staging at a single tertiary care center between January 2010 and June 2016, and who had also undergone unilateral bone marrow biopsy of the iliac crest, were eligible for inclusion in this Health Insurance Portability and Accountability Act (HIPAA)-compliant, retrospective study that was approved by the Institutional Review Board of Memorial Sloan Kettering Cancer Center (protocol no. 18–175); informed consent was waived. Additional inclusion criteria were the following: availability of pathology reports on bone marrow biopsy specimens, containing information on the bone marrow cellularity (in %) as well as, in case of lymphoma involvement, the percentage of bone marrow cellularity involved (in %); clinical and laboratory data, including age, white blood cell count (WBC; upper normal limit, 11.0 x10^9^/L), lactate dehydrogenase levels (LDH; upper normal limit, 246 U/L), and Ki-67 proliferation index (in %), obtained within two weeks of the pre-therapeutic PET/CT. Patients with blood glucose levels > 180 mg/dL, as well as patients not examined with one of the four pre-specified PET/CT scanners listed below, were excluded. The study was approved by the local institutional review board of Memorial Sloan Kettering Cancer Center (IRB no. 18-175); informed consent was waived due to the retrospective design of the study.

### 4.2. Bone Marrow Histology—Percentage of Involvement

All biopsy samples were evaluated by a senior board-certified reference pathologist in accordance with the 2016 revision of the World Health Organization classification of lymphoid neoplasms [41]. Flow cytometry and fluorescence in situ hybridization (FISH) analyses were also taken into account in patients for whom the respective tests were available. In patients with bone marrow involvement demonstrated by the latter tests as well as by histology, percentage of involvement was obtained from the histology report. In addition to the percentage of cellular (“red”) bone marrow infiltrated by lymphoma cells (i.e., the relative percentage of involvement; termed REL), the percentage of absolute bone marrow involvement (i.e., with regard to the entire marrow space, including cellular and fatty marrow; termed ABS) was calculated as the product of bone marrow cellularity (in %) and REL. This was done because PET image analysis (see below) included the entire pelvic bone marrow volume, rather than just the cellular bone marrow. A 5% and 10% threshold of bone marrow involvement were applied, separately for REL and ABS, to enable pairwise classification (see below).

### 4.3. Imaging Protocol

PET/CT, covering the anatomy from the mid skull to upper thigh, was performed approximately 60 min after intravenous administration of 12–15 mCi of [18F]FDG. Patients had fasted for more than 6 h. PET was performed in three-dimensional mode, with at least 3 min per bed position, and a voxel size of 5.5 × 5.5 × 3.3 mm³, using one of four different PET/CT scanner models from the GE Healthcare Discovery line: STE, 600, 690, 710 (GE Healthcare, Waukesha, WI, USA). Spiral CT without contrast was performed with a tube current of 60 mAs, a tube voltage of 120–140 kVp, and a 5 mm section thickness, and was used for PET attenuation correction and anatomical correlation.

### 4.4. Image Analysis and Radiomic Feature Analysis

Using the Beth Israel PET/CT viewer plugin for FIJI [42], the metabolic tumor volume (MTV) of the bony pelvis was semi-automatically constructed, using the previously recommended 41% SUVmax threshold [43] (Figure 2). The co-registered CT component of PET/CT was used to ensure correct matching of MTVs and the underlying anatomy, and to manually adjust the segmented MTVs in case of difficulties in separation of the pelvic bones from the adjacent structures, such as bowel loops and muscles that may show increased [18F]FDG uptake. Based on the MTVs, a three-dimensional analysis was performed, which comprised the calculation of the three frequently used maximum, mean and peak standardized uptake values (SUVmax, SUVmean, and SUVpeak), as well as calculation of the following 16 second-order 3D texture features derived from the gray-level co-occurrence matrix (GLCM), following intensity discretization to a fixed bin width of 25: Entropy; Homogeneity; Contrast; Correlation; Angular second moment; Difference entropy; Difference variance; Inverse difference moment; Sum average; Sum entropy; Sum variance; Cluster prominence; Cluster shade; Maximum probability; and two Informational measures of correlation [44]. To correct for a possible influence of the technical differences between the four PET/CT scanners, ComBat harmonization was performed, as previously described [45].

### 4.5. Image Analysis and Radiomic Feature Analysis

Prior to classification, reduction of dimensionality of the radiomic features (including SUVs and texture features) using principle component analysis (PCA) was performed to reduce the degree of data redundancy (i.e., correlations between individual features). Principle components were extracted based on Eigenvalues > 1, with a maximum of 25 iterations for convergence. Principal radiomic components constituted the so-called “radiomic signature” that was used for each of the following pairwise comparisons:

Involvement vs. no involvement 

REL ≤ 5% vs. REL > 5% (where REL ≤ 5% comprises involvement of 0–5%) 

REL ≤ 10% vs. REL > 10%

ABS ≤ 5% vs. ABS > 5% (where ABS ≤ 5% comprises involvement of 0–5%) 

ABS ≤ 10% vs. ABS > 10%

In addition, in patients with confirmed bone marrow involvement, the radiomic signature was tested versus the SUVs regarding their ability to distinguish between low (<30%) and high (≥30%) Ki-67 proliferation indices.

### 4.6. Machine Learning for Prediction of Bone Marrow Involvement

The three SUVs and, separately, the principal radiomic components generated in the previous step were used as input for a multi-layer perceptron, feed-forward, artificial neural network (MLP-NN), which uses a backpropagation learning algorithm [46], to determine their value for prediction of bone marrow involvement and high/low Ki-67. For each pairwise classification, 70% of the cases were randomized to the training dataset, and the remaining 30% to the test dataset. Since the starting point of the neural network is an initial guess at the weights of the individual principal radiomic components, the classification step was performed five times for each pair (e.g., REL ≤ 5% vs. REL > 5%; or Ki-67 < 30% vs.Ki-67 ≥ 30%), separately for SUVs and radiomic principal components. A minimum of one hidden layer (activation function: hyperbolic tangent), with a minimum of three neurons per hidden layer, were used for the MLP neural network (output activation function: softmax). 

Following the above described, purely PET-based analyses, the classification step for bone marrow involvement was repeated, again five times, for each pairwise comparison, but this time using the radiomic principal components together with (continuous) WBC and LDH levels as input variables. This was done to determine whether the integration of PET radiomic and routine laboratory data could improve prediction of bone marrow involvement in MCL. 

Areas under the ROC curves (AUCs) and classification accuracies for training and test datasets were used as main outcome measures. All statistical tests, including the MLP neural network analysis, were performed using IBM SPSS 24.0 (IBM Corp., Armonk, NY, USA).

## 5. Conclusions

In conclusion, our study results suggest that addition of [18F]FDG-PET radiomic texture features to SUVs clearly improves the assessment of bone marrow involvement in MCL, and also PET-based evaluation of cell proliferation. The performance of the radiomic signature that includes both PET texture features and SUVs appears to be enhanced by addition of routine laboratory data, especially in cases of very low-level infiltration with lymphoma cells. Nevertheless, for the clinical application of this technique, further improvement is required, possibly by addition of radiomic features extracted from CT, or, in the case of PET/MRI, from one or more MRI pulse sequences. Evaluation of changes in radiomic features during and after treatment, with repeat biopsy results serving as the reference standard, would be another area of clinical interest. Finally, further validation in external cohorts is required to confirm the results of our study. 

## Figures and Tables

**Table 1:** Baseline demographic, clinical, laboratory and biological data of the entire cohort and the training and test cohorts for involved vs. uninvolved bone marrow.

Characteritsic	Entire Population (97 Patients)	Training Cohort (68 Patients)	Test Cohort (29 Patients)
**Age**	63.5 ± 12.5	64.3 ± 12.8	61.6 ± 11.8
**Female**	31/97 (32.0%)	20/68 (29.4%)	11/29 (37.9%)
**Ann Arbor Stage**			
I–II	14/97 (14.4%)	12/68 (17.6%)	2/29 (6.9%)
II–IV	83/97 (85.6%)	56/68 (82.4%)	27/29 (93.1%)
**Blastoid differentiation**	21/97 (21.6%)	15/68 (22.1%)	6/29 (20.7%)
Blastic	18/97 (18.6%)	12/68 (17.6%)	6/29 (20.7%)
Pleomorphic	3/97 (3.1%)	3/68 (4.4%)	0/29 (0%)
**WBC** (×10^9^/L)	10.5 ± 11.9	10.2 ± 12.4	10.7 ± 11.0
**LDH** (U/L)	232.3 ± 86.0	236.4 ± 97.2	222.4 ± 51.4
**ECOG** ≥ 2	9/97 (9.3%)	6/68 (8.8%)	3/29 (10.3%)
**Bone marrow involvement**	67/97 (69.1%)	47/67 (70.1%)	20/29 (70.0%)
REL	33.0 ± 29.1%	33.0 ± 29.6%	32.9 ± 28.6%
ABS	22.6 ± 23.6%	24.5 ± 22.6%	21.8 ± 22.0%
Ki-67	28.9 ± 23.7%	29.3 ± 24.6%	28.0 ± 22.0%

WBC, white blood count; LDH, lactate dehydrogenase; ECOG, Eastern Cooperative Oncology Group Performance Status; REL, percentage of involvement of cellular bone marrow; ABS, percentage of involvement relative to the entire marrow space.

**Table 2:** Mean classification accuracies for radiomics only and radiomics + laboratory data.

Metrics	Training Accuracy Median (Range) %	Test Accuracy Median (Range) %	AUC Median (Range) %
**SUVs:**			
BMB pos. vs. BMB neg.	69.1 (69.1–70.6)	69.0 (69.0–69-0)	0.61 (0.60–0.66)
REL ≤ 5% vs. REL > 5%	54.4 (51.5–58.8)	72.4 (69.0–72.4)	0.68 (0.68–0.68)
REL ≤ 10% vs. REL > 10%	69.1 (61.8–70.6)	69.0 (65.5–72.4)	0.69 (0.68–0.69)
ABS ≤ 5% vs. ABS > 5%	61.8 (61.8–66.2)	72.4 (69.0–75.9)	0.70 (0.69–0.73)
ABS ≤ 10% vs. ABS > 10%	69.1 (67.6–70.6)	89.7 (86.2–89.7)	0.75 (0.74–0.75)
**Radiomics signature:**			
BMB pos. vs. BMB neg.	70.6 (69.1–72.1)	72.4 (69.0–72.4)	0.68 (0.61–0.73)
REL ≤5 % vs. REL > 5%	70.6 (66.2–76.5)	79.3 (79.3–82.8)	0.77 (0.75–0.84)
REL ≤ 10% vs. REL > 10%	76.5 (72.1–76.5)	75.9 (72.4–79.3)	0.80 (0.79–0.85)
ABS ≤ 5% vs. ABS > 5%	73.5 (67.6–73.5)	72.4 (69.0–79.3)	0.81 (0.77–0.82)
ABS ≤ 10% vs. ABS > 10%	79.4 (77.8–82.4)	82.8 (79.3–86.2)	0.85 (0.83–0.86)
**Radiomics + Laboratory:**			
BMB pos. vs. BMB neg.	76.5 (71.2–82.4)	72.4 (69.0–75.9)	0.76 (0.71–0.81)
REL ≤ 5% vs. REL > 5%	70.6 (67.6–72.1)	82.8 (79.3–93.1)	0.82 (0.81–0.84)
REL ≤ 10% vs. REL > 10%	77.9 (71.1–80.9)	75.9 (72.4–79.3)	0.84 (0.82–0.87)
ABS ≤ 5% vs. ABS > 5%	75.0 (70.6–79.4)	75.9 (72.4–79.3)	0.81 (0.79–0.83)
ABS ≤ 10% vs. ABS > 10%	79.4 (72.1–80.9)	79.3 (75.0–82.8)	0.84 (0.82–0.86)

BMB, bone marrow biopsy; REL, percentage of involvement of cellular bone marrow; ABS, percentage of involvement relative to the entire marrow space.

## Supplementary Materials

The following are available online at https://www.mdpi.com/2072-6694/12/5/1138/s1: Table S1. Component matrix showing the contributions of the individual radiomic features to the five principal components that formed the radiomic signature.
