# Supplementary material for: [18F]FDG-PET/CT Radiomics for Prediction of Bone Marrow Involvement in Mantle Cell Lymphoma: A Retrospective Study in 97 Patients
Source: Cancers (Basel). 2020 May 2;12(5):1138. doi: 10.3390/cancers12051138 (PMC7281173; doi:10.3390/cancers12051138)
Supplement: Supplementary file 1 [file cancers-12-01138-s001.pdf]

Supplementary Materials: [18F]FDG-PET/CT Radiomics for Prediction of Bone Marrow Involvement in Mantle Cell Lymphoma: A Retrospective Study in 97 Patients

Marius E. Mayerhoefer, Christopher C. Riedl, Anita Kumar, Ahmet Dogan, Peter Gibbs, Michael Weber, Philipp B. Staber, Sandra Huicochea Castellanos and Heiko Schöder

Table 1. Component matrix showing the contributions of the individual radiomic features to the five principal components that formed the radiomic signature.

Radiomic feature	Principal Component				
	**1**	**2**	**3**	**4**	**5**
SUVmean	0.409	-0.545	0.693	-0.154	0.092
SUVpeak	0.319	-0.518	0.769	-0.133	0.061
SUVmax	0.272	-0.635	0.700	-0.134	0.021
entropy	0.970	0.173	-0.068	0.067	0.074
homogeneity	-0.877	0.389	0.169	-0.123	0.073
contrast	0.835	-0.453	-0.101	0.185	-0.189
angular second moment	-0.914	-0.207	0.072	0.072	-0.207
difference entropy	0.822	-0.473	-0.177	0.212	-0.124
difference variance	0.764	-0.516	-0.107	0.238	-0.231
correlation	-0.084	0.924	0.203	0.007	0.131
cluster prominence	0.513	0.637	0.358	0.374	-0.075
cluster shade	-0.166	0.436	0.407	0.760	0.032
measure of correlation 1	-0.872	0.409	0.166	-0.125	0.056
measure of correlation 2	0.402	-0.066	-0.300	0.141	0.697
inverse difference moment	0.005	0.940	0.271	-0.012	-0.028
maximum probability	-0.869	-0.182	0.165	0.251	-0.202
sum average	0.833	0.478	0.017	-0.243	-0.030
sum entropy	0.784	0.594	0.080	0.032	0.075
sum variance	0.814	0.498	0.065	-0.203	-0.074
variance	0.822	0.508	0.076	-0.155	-0.074
sphericity	-0.139	-0.452	0.079	0.136	0.628
